# Long-Term Management of Recurrent Cholecystitis after Initial Conservative Treatment: Endoscopic Transpapillary Gallbladder Stenting

**DOI:** 10.1155/2018/3983707

**Published:** 2018-04-12

**Authors:** Hideki Kamada, Hideki Kobara, Naohito Uchida, Kiyohito Kato, Takayuki Fujimori, Kiyoyuki Kobayashi, Takuma Yamashita, Masahiro Ono, Yuichi Aritomo, Kunihiko Tsutsui, Keiichi Okano, Yasuyuki Suzuki, Tsutomu Masaki

**Affiliations:** ^1^Department of Gastroenterology and Neurology, Faculty of Medicine, Kagawa University, Kagawa, Japan; ^2^Department of Gastroenterological Surgery, Faculty of Medicine, Kagawa University, Kagawa, Japan

## Abstract

**Background:**

Endoscopic transpapillary gallbladder stenting (ETGBS) is an effective procedure for treating high-risk patients with acute cholecystitis and severe comorbidities. However, the efficacy of ETGBS for recurrent cholecystitis (RC) remains unclear. This study aimed to explore its efficacy in patients with RC for whom cholecystectomy is contraindicated because of its high surgical risk.

**Methods:**

Data on 19 high-risk patients who had undergone ETGBS for RC after initial conservative therapy in our institution between June 2006 and May 2012 were retrospectively examined. The primary outcome was the clinical success rate, which was defined as no recurrences of acute cholecystitis after ETGBS until death or the end of the follow-up period. Secondary outcomes were technical success rate and adverse events (AEs).

**Results:**

The clinical success rate of ETGBS was 100%, the technical success rate 94.7%, and AE rate 5%: one patient developed procedure-related mild acute pancreatitis. The clinical courses of all patients were as follows: four died of nonbiliary disease, and the remaining 15 were subsequently treated conservatively. The median duration of follow-up was 14.95 months (range 3–42 months).

**Conclusions:**

ETGBS is an effective alternative for managing RC in high-risk patients with severe comorbidities.

## 1. Introduction

Laparoscopic cholecystectomy is currently the standard treatment for patients with early stage acute cholecystitis [1, 2]. Cholecystectomy is a commonly performed and safe procedure; however, invasive surgery is sometimes contraindicated in high-risk patients with complex comorbidities such as severe coagulopathy or poor performance status [3]. Moreover, although percutaneous transhepatic gallbladder aspiration/drainage (PTGBA/D) is considered a temporary therapy aimed at decompressing the gallbladder, the percutaneous transhepatic approach is controversial in patients at high risk of intra-abdominal bleeding or of removing their drainage tubes themselves [4–7]. Thus, endoscopic transpapillary gallbladder drainage (ETGBD) has recently been proposed as an alternative procedure for high-risk patients with acute cholecystitis [8–12]. ETGBD generally involves endoscopic nasotranspapillary gallbladder drainage and endoscopic transpapillary gallbladder stenting (ETGBS) without risks of tube self-withdrawal. In particular, ETGBS has been adopted as the initial approach in high-risk patients with acute cholecystitis or in patients with end-stage liver disease related to advanced cancer or those awaiting liver transplantation [13–19]. However, ETGBS has a mild risk of related pancreatitis due to the transpapillary approach, and conservative therapy may sometimes be curative for acute cholecystitis without the need for additional therapy. Accordingly, ETGBS may be a suitable option for recurrent cholecystitis (RC) after conservative treatment with antibiotics or PTGBA/D. Few studies have reported the long-term clinical outcomes of ETGBS for the prevention of RC. Hence, this retrospective study aimed to assess the clinical efficacy and long-term outcomes of ETGBS after initial conventional therapy in surgically high-risk patients with RC.

## 2. Methods

### 2.1. Patients

This retrospective study was conducted at a single center, Kagawa University Hospital. Data on 19 high-risk patients who had undergone ETGBS for RC after initial conservative therapy in our institution between June 2006 and May 2012 were retrospectively examined. Patient characteristics, including age, comorbidities (benign and malignant), physical status, presence of dementia, severity of cholecystitis, and therapy prior to ETGBS, were collected. The inclusion criteria were high-risk patients with RC within 2 months after conservative treatment with antibiotics or PTGBA/D for first episodes of acute cholecystitis for whom cholecystectomy was contraindicated because of its high surgical risk. High-risk patients with acute cholecystitis were defined as follows: (1) patients with increased postoperative morbidity and mortality, such as those with cirrhosis, cerebral disease, cardiopulmonary disease, malignancy, or other significant medical illnesses; (2) those with severe coagulopathy or thrombocytopenia; (3) patients with anatomically inaccessible gallbladder or other anatomic abnormalities; (4) those with a large amount of ascites, which is a known contraindication to percutaneous therapy.

ETGBS was performed with the aim of permanently managing RC. Scheduled stent exchanges were not performed in this study; however, patients were prospectively followed for stent exchange at the discretion of the endoscopist, surgical intervention, or death.

Acute cholecystitis was diagnosed based on (1) presence of symptoms, (2) abnormalities of relevant laboratory data (white blood cell count and C-reactive protein), and (3) a thickened gallbladder wall, pericholecystic fluid, and a distended gallbladder shown by imaging studies (transabdominal ultrasonography and computed tomography). The severity of acute RC was graded according to the Tokyo Guidelines (TG) grading system [20]. American Society of Anesthesiologists (ASA) scores were used to denote the patients' preoperative physical health status. All patients with acute or RC had been treated under fasting with antibiotics and intravenous administration of lactated Ringer's solution.

This study was approved by the Clinical Ethics Committee of Kagawa University Hospital. All patients had provided written informed consent to undergo the ETGBS procedures.

### 2.2. ETGBS Procedure

**Figure 1:**
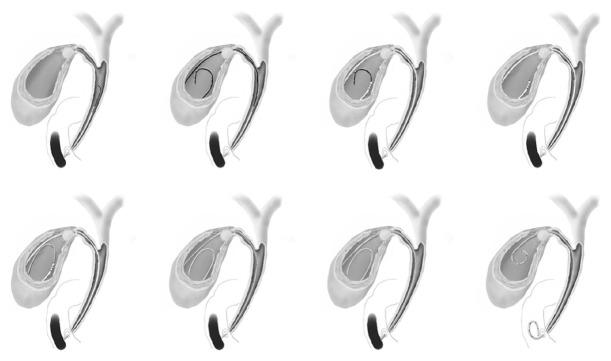
Depiction of the procedure for endoscopic stenting of the common bile duct and gallbladder. A catheter is inserted deep into the bile duct and a guidewire advanced into the cystic duct and gallbladder. The catheter is inserted over the guidewire up to the fundus of the gallbladder. A double-pigtail polyethylene stent is then inserted.

**Figure 2:**
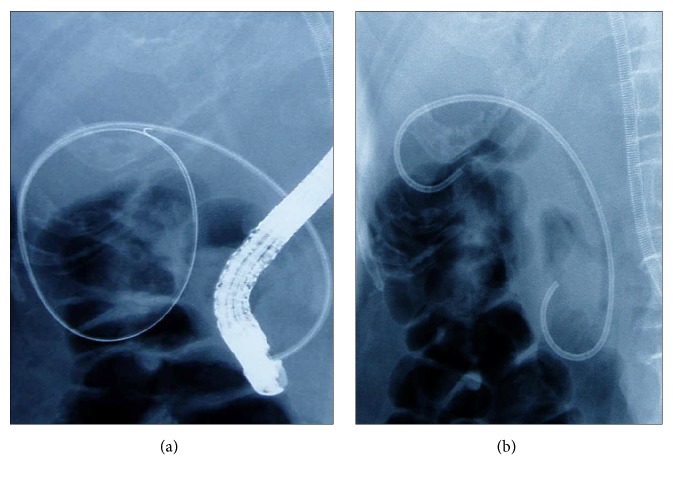
(a) Fluoroscopic image of a guidewire coiled in the gallbladder. (b) Fluoroscopic image of a stent extending from the duodenum into the gallbladder.

ETGBS was performed as previously described [12] and as shown in Figure 1. All patients were placed in a prone position before undergoing endoscopic retrograde cholangiopancreatography (ERCP) with side-viewing endoscopes (TJF240 or TJF260V; Olympus, Tokyo, Japan) after sedation with intravenous midazolam (0.05 mg/kg). Selective bile duct cannulation was achieved by advancing an ERCP catheter (MTW, Düsseldorf, Germany) over a 0.025- or 0.032-inch hydrophilic guidewire (e.g., Radifocus; Terumo, Tokyo, Japan) into the cystic duct and gallbladder. After the catheter had been inserted over the guide wire into the fundus of the gallbladder (Figure 2(a)), a stiff guide wire was substituted for the hydrophilic guidewire (VisiGlide; Olympus, Tokyo, Japan, or Hydra Jagwire; Boston Scientific Japan, Tokyo, Japan). A 7F, 10 to 15 cm long, double-pigtail polyethylene stent (Olympus, Tokyo, Japan) was then deployed (Figure 2(b)), crossing the ampulla with the proximal and distal pigtails in the gallbladder and duodenum, respectively. The length of the stent was determined by pulling the guide wire out of the gallbladder to the ampulla and measuring that distance.

### 2.3. Outcome Measures

The primary outcome was clinical success rate. Secondary outcomes were the rates of technical success and procedure-related adverse events (AEs). Clinical success was defined as no recurrences of acute cholecystitis after ETGBS until death or the end of the follow-up period. Technical success was defined as successful placement of a double-pigtail stent in the gallbladder. Procedure-related AEs were graded according to the American Society for Gastrointestinal Endoscopy lexicon's grading system [24].

## 3. Results

**Figure 3:**
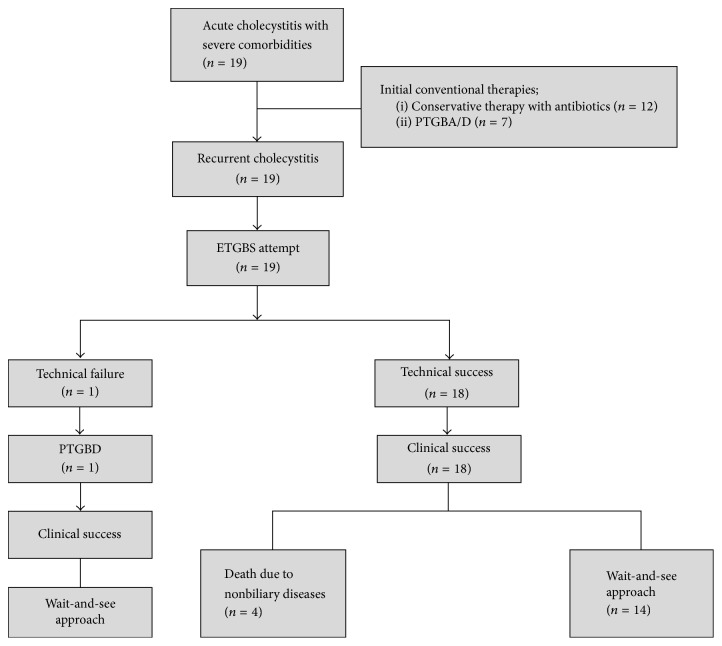
Flow diagram of patient's clinical courses.

Data of 19 consecutive patients (10 men, 9 women; median age of 84 years; range 60–93 years) were examined. Detailed patient characteristics are summarized in Table 1. All included patients had severe comorbidities, 15 (78.9%) of them having severe dementia. ASA scores were used to assess the patients' preoperative physical status: 15 patients (78.9%) were classified as having Class 4 status, three (15.8%) Class 3, and one (5.3%) Class 2, contraindicating cholecystectomy. The severity of acute cholecystitis according to TG13 grades was as follows: four patients (21.1%) were classified as having Grade I acute cholecystitis and 15 patients (78.9%) as having Grade II. Prior to ETGBS, 12 patients had received conservative therapy with antibiotics and seven had undergone PTGBA/D. Outcomes are shown in Table 2. ETGBS was technically successful in 18 (94.7%) of the 19 patients. ETGBS failed in one patient, after which PTGBD was performed. Endoscopic sphincterotomy (EST) was performed to remove common bile duct stones in three patients. The clinical success rate was 100% in the 18 patients for whom ETGBS had been successful. Although no severe procedure-related AEs occurred, one patient developed mild acute pancreatitis; thus the AE rate was 5.3%. The clinical courses of all patients were as follows. Four patients died of nonbiliary diseases, namely, malignant lymphoma (3 months after ETGBS), chronic respiratory failure (9 months after ETGBS), aspiration pneumonitis (17 months after ETGBS), and cerebral infarction (42 months after ETGBS). The remaining 15 patients, including the one in whom ETGBS was unsuccessful, were thereafter managed conservatively.  Figure 3 is a flow diagram showing the patients' clinical courses. The median duration of follow-up was 14.95 months (range 3–42 months). Although spontaneous distal migration of the stent occurred in one patient 23 months after ETGBS, no patients who had undergone ETGBS required stent removal or exchange during follow-up. None of the included patients received oral antibiotics and biliary medicines such as ursodeoxycholic acid during follow-up after recovering from acute cholecystitis.

## 4. Discussion

ETGBS is considered an effective treatment option for acute cholecystitis in poor surgical candidates with severe comorbidities. However, there are few published data on long-term outcomes in terms of symptomatic cholelithiasis in such patients. Because advanced skills are required to perform ETGBS and there is a risk of procedure-related severe pancreatitis because of the transpapillary approach, conventional measures such as conservative therapy or PTGBA/D are generally selected as the initial approach for managing acute cholecystitis. Thus, in the present study, ETGBS was performed for RC occurring after such initial therapies rather than for the first episode of acute cholecystitis. This is the first study demonstrating that ETGBS is a reasonable option for long-term management of RC after initial conventional therapy.

Cholecystectomy is the mainstay of management of acute cholecystitis [1, 2]. However, the mortality rate of emergency cholecystectomy (approximately 30%) is still unsatisfactory in high-risk patients with severe comorbidities such as liver cirrhosis, serious cardiopulmonary disease, or significant medical illness [21]. Therefore, such patients may be treated with conservative therapy with antibiotics or PTGBA/D as a temporary means of decompressing the gallbladder. Reported technical success rates for PTGBA and PTGBD range between 82%–97% and 97%–100%, respectively [4–7]. However, PTGBA/D procedures are sometimes contraindicated by factors such as severe coagulopathy, severe thrombocytopenia, anatomically inaccessible lesions, or the presence of ascites [7]. Recently, ETGBD has been reported as an alternative to percutaneous gallbladder drainage for managing acute cholecystitis in such patients. Since Kozarek introduced endoscopic transpapillary cannulation of the gallbladder in 1984 [25], several endoscopists, including our senior colleagues [12], have developed ETGBD techniques for treating acute cholecystitis. However, ETGBD has the limitation that older persons with dementia are at risk of removing their drainage tubes themselves and these tubes cannot be implanted in the gallbladder long term. Thus, ETGBD is best considered a temporary and bridge therapy to surgery. Consequently, there is a need for an effective long-term therapeutic strategy for managing acute cholecystitis in high-risk patients who are poor candidates for emergency cholecystectomy. Recently, several authors have reported that ETGBS is an effective long-term option for managing acute cholecystitis in high-risk patients [17, 21, 22]. Previously reported outcomes of ETGBS are shown in Table 3 [8, 13–17, 21–23]. The mean overall clinical success rate was 92.6% (range 64%–100%) (216 cases), including our results. Although there are few studies on long-term clinical courses, our data on patients undergoing ETGBS demonstrate excellent long-term outcomes with a clinical success rate of 100% in 19 patients in whom the procedure was technically successful. None of our patients required stent removal or exchange, likely because stents have the following favorable effects. First, stents prevent impaction or migration of gallstones into the cystic duct. Second, even occluded stents may still provide drainage of the gallbladder because bile may flow around them. Third, bile flow may improve because the cystic duct is straightened by the placement of the stent. However, more studies are needed to clarify the reasons for the excellent patency results.

The reported technical success rate of ETGBS ranges from 79.3% to 100%, as shown in Table 3. In our study, the technical success rate of ETGBS (94.7%) was similar to that in previous studies. One limitation of ETGBD/S is that it can be technically difficult to perform in some patients, considerable skill being required to pass the guidewire through the cystic duct. Several factors can contribute to failure, namely, severe inflammatory or malignant strictures, obstruction of the neck of the gallbladder by impacted stones, tortuosity of the duct, and cystic duct outlet not visible on the cholangiogram. These high rates of technical success may be attributable to the level of skills gained by performing many of these procedures.

As shown in Table 3, the mean overall rate of AEs was 2.5% in 216 cases; there were no serious AEs. In our series, there was only one case of postprocedure mild pancreatitis. However, although ETGBS is thus relatively safe, a careful approach is needed to prevent post-ERCP. Moreover, great care should be taken to avoid perforation of the cystic duct or gallbladder. Pannala et al. reported that biliary perforation occurred in 2% of patients who underwent this procedure [26]. Other AEs, including migration of the stent and cholangitis, have been reported. In 20 patients with long-term follow-up (median 606.5 days) after ETGBS, Lee et al. [21] reported that spontaneous distal migration occurred in two patients 7 months after ETGBS with EST and that one patient who had undergone ETGBS without EST developed cholangitis with choledocholithiasis 19 months after the procedure. In our study, spontaneous distal migration of a stent occurred in one patient 23 months after ETGBS without EST; there were no complications and no additional interventions were required.

Jang et al. recently reported that endoscopic ultrasound-guided transmural gallbladder drainage (EUS-GBD) is comparable with PTGBD for nonsurgical patients with comorbidities in terms of technical feasibility, efficacy, and safety [27]. In contrast, the consensus report of Tokyo Guidelines 13 reported that EUS-GBD results in a relatively high incidental rate of AEs of 11%–33% [28]. Because severe and uncontrolled complications such as bile leakage, stent migration into the gallbladder or intra-abdominal space, deviation of the stent from the gallbladder, puncture-induced hemorrhage, and perforation of the peritoneum are expected, EUS-GBD has not yet been established as a standard therapy for gallbladder drainage. Further research is needed to determine whether EUS-GBD or ETGBS is superior.

Finally, first-line therapy for acute cholecystitis in high-risk patients should be discussed to establish a therapeutic strategy. Performing ETGBS requires advanced skills and has the potential risk of procedure-related severe pancreatitis because of the transpapillary approach. Regarding the rate of recurrence of acute cholecystitis, Ha et al. reported that 1-year and 3-year recurrence rates of in-patients who did not undergo subsequent cholecystectomies were 35% and 46% [29], respectively. Schmidt et al. reported a recurrence rate of 24% [30]. In other words, conservative approaches are appropriate for 50%–70% of all patients treated for acute cholecystitis. Therefore, we recommend conventional therapies such as conservative treatment or PTGBA/D for the initial approach to acute cholecystitis. After such initial therapy, ETGBS should be considered for long-term management when RC occurs. However, no data are available on how long stents remain patent or when and whether stent removal and exchange are necessary. A large study is needed to resolve these issues.

In conclusion, ETGBS may be an effective and reasonable option for long-term management of recurrent cholecystitis after initial conventional therapies in high-risk patients who are poor candidates for cholecystectomy.

## Figures and Tables

**Table 1:** Patient characteristics.

Characteristics	Value
Number of patients, *n*	19
Sex, male/female, *n*.	10/9
Age, median (range), yr	84 (60–93)
Comorbidities, *n*	
Benign	
Poor cerebral condition	8
Poor cardiovascular condition	2
Poor cerebral and cardiovascular condition	2
Poor pulmonary function	1
Ulcerative colitis	1
Bedridden due to gonarthrosis	1
Multiorgan failure	1
Malignant	
Bile duct cancer	1
Gastric cancer	1
Lymphoma	1
ASA class	
I	0
II	1
III	3
IV	15
Dementia	
YES	15
NO	4
Severity grading per Tokyo guidelines 2013	
I	4
II	15
First therapy prior to ETGBS	
Conservative with antibiotics	12
PTGBA/D	7

F, female; M, male.

**Table 2:** Outcomes of endoscopic transpapillary gallbladder stenting (*n* = 19).

Primary outcome	Clinical success rate, % (*n*)	100 (18/18)
Secondary outcomes	Technical success rate, % (*n*)	94.7 (18/19)
	Procedure-related adverse events rate, % (*n*)	5.3 (1/19)^#^

^#^Mild acute pancreatitis.

**Table 3:** Published outcomes of endoscopic transpapillary gallbladder stenting.

Author (year) [Ref.]	Type of study	Number of cases	Technical success rate (%)	Clinical success rate (%)	Rate of adverse events (%)	Follow-up, months	Number of relapses
Tamada et al. (1991) [8]	R	14	100	64	0	2	0
Kalloo et al. (1994) [13]	R	4	100	100	0	11–17	0
Gaglio et al. (1996) [14]	R	3	100	100	0	4–6	2
Shrestha and Lasch (2001) [15]	R	13	100	100	0	1–36	1
Conway et al. (2005) [16]	R	29	89.7	97	0	9.4	2
Schlenker et al. (2006) [17]	R	23	100	78.3	0	2–54	3
Lee et al. (2011) [21]	P	29	79.3	100	17.2	20	2
Maekawa et al. (2013) [22]	R	46	80.4	96.8	0	1–60	1
Itoi et al. (2015) [23]	P	36	86.1	90.3	2.7	1	0
Our study	R	19	94.7	100	5.3	3–42	0

Total		216	93.6	92.6	2.5		

P, prospective; R, retrospective.
